# Ultrasound Findings of Plasma Leakage in Dengue Fever

**DOI:** 10.4269/ajtmh.18-0422

**Published:** 2018-12

**Authors:** Sachita Shah, Robert Rolfe, German Henostroza, Carlos Seas

**Affiliations:** 1 Department of Emergency Medicine, University of Washington School of Medicine and Harborview Medical Center, Seattle, Washington;; 2 Department of Medicine, University of Alabama, Birmingham, Alabama;; 3 Division of Infectious Diseases, Department of Medicine, University of Alabama, Birmingham, Alabama;; 4 Department of Tropical and Infectious Diseases, Cayetano Heredia National Hospital, Lima, Peru

**Figure 1.**
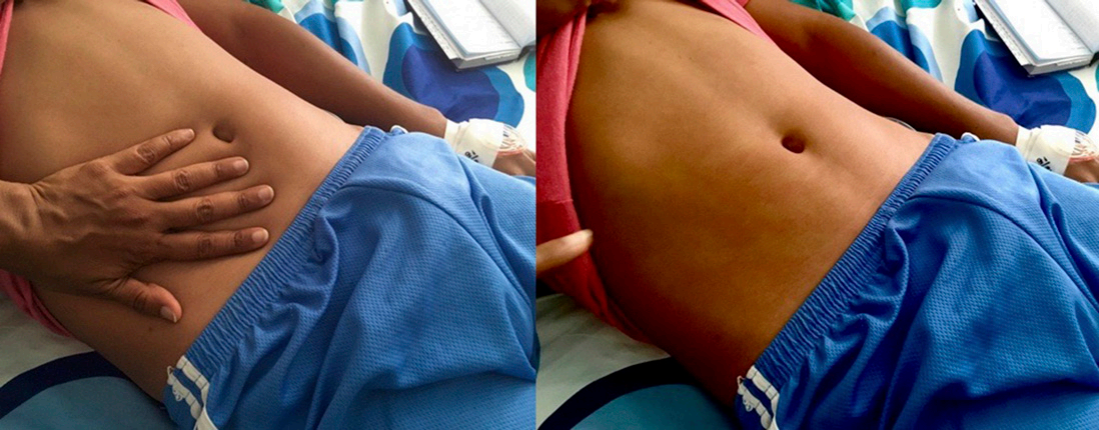
Characteristic maculopapular rash of Dengue fever. Note the outline of the palm after brief pressure applied indicating blanching. This figure appears in color at www.ajtmh.org.

A 26-year-old male from Iquitos, Peru (urban, peri-jungle), presented with a 5-day history of fevers, headaches, and body aches. Three days before presentation, the patient started having diffuse abdominal pain, later localizing to the epigastrium with associated nausea, vomiting, and scant hematemesis. He also had increasing restlessness, insomnia, and constipation. Physical examination revealed temperature 36.8°C, heart rate 68 beats per minute, respiratory rate 21, and blood pressure 130/90. He was ill-appearing, with a diffuse blanching maculopapular rash, sparing the palms and soles, without evidence of petechiae (Figure 1). Cardiovascular and pulmonary examinations were unremarkable without evidence of dullness to percussion or crackles. Abdominal examination was notable for mild right upper quadrant and epigastric tenderness to palpation, without hepatosplenomegaly. He had no peripheral edema. Laboratory values were notable for a white blood cell count of 3,900 cells/mm^3^, hematocrit 45%, and platelets of 27,000 cells/mm^3^. Transaminases were aspartate aminotransferase 208 (< 40 IU/L), alanine aminotransferase 124 (< 40 IU/L), and total bilirubin 1.1/dL mg/dL. Thick blood smears for malaria were negative. NS1 test taken on the day of admission was positive for dengue virus.

**Figure 2.**
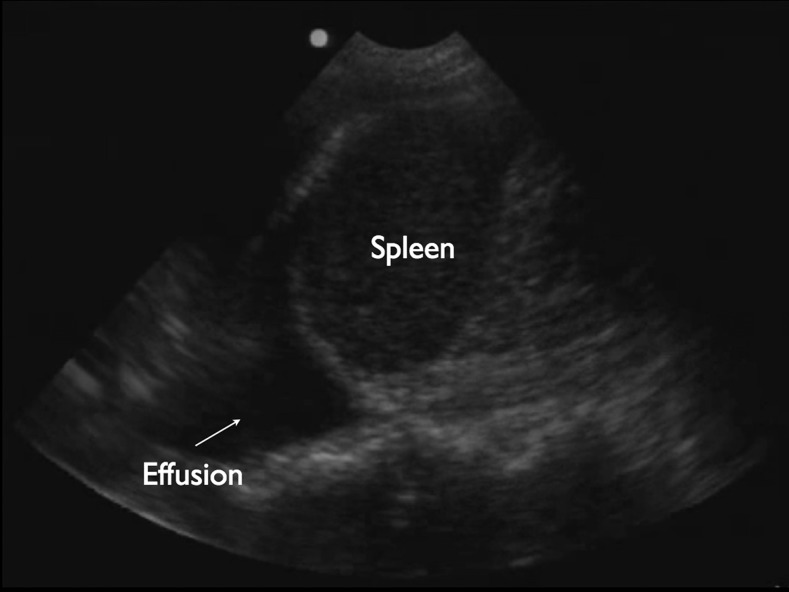
Ultrasound image of left pleural effusion. Simple pleural fluid appears black, or hypoechoic, on ultrasound in the costophrenic recess (arrow). Sonographic evidence of sub-clinical third-spacing of fluid in Dengue fever can be seen even without physical exam findings consistent with pleural fluid.

**Figure 3.**
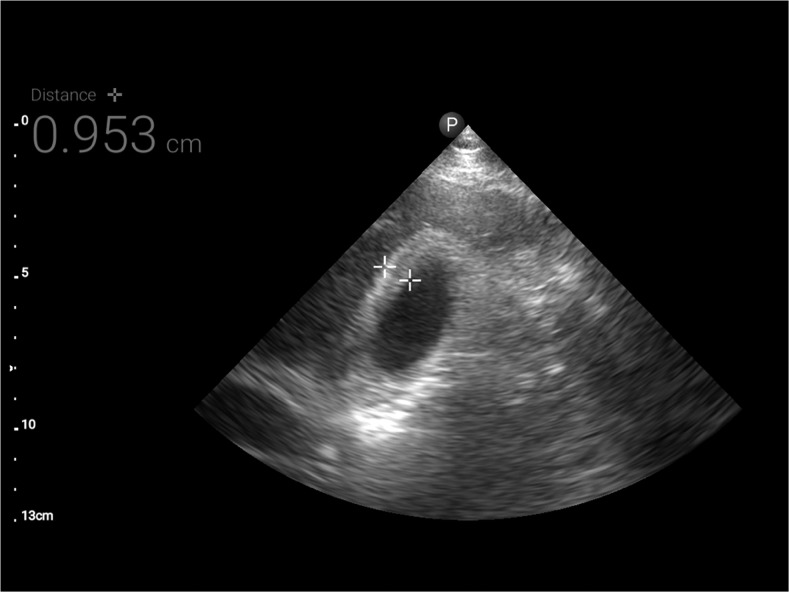
Ultrasound image showing gall bladder wall thickening of 0.93 cm (normal < 0.3 cm).

Bedside, point-of-care ultrasound of the right upper quadrant and bilateral chest was performed during the convalescent phase (day 7 of illness) (Philips Lumify ultrasound with curved, abdominal probe), revealing a uniformly thickened gallbladder wall measuring 0.93 cm (Figures 2 and 3). No ascites, biliary stones, or sonographic Murphy’s sign was present. The patient was also found to have a scant amount of fluid in the left pleural space.

Third spacing of fluid in various areas of the body can occur in the critical (plasma leak) phase of dengue, manifesting in subtle effusions and gallbladder wall congestion that may not be evident on physical examintation. Point-of-care, clinician-performed ultrasound adds value in the evaluation of many tropical infectious diseases, allowing clinicians access to early findings that may guide initial diagnosis and management.^1^ Ultrasound findings of severe dengue include pleural and pericardial effusions, ascites, gallbladder wall thickening, and diffuse peripheral edema.^2^ Gallbladder wall thickening is nonspecific; however, a uniform pattern of thickening has been associated with less severe dengue fever, as seen in our patient, whereas a honeycomb pattern has been found to be more prevalent in severe dengue fever. Changes in uniform thickening to a more heterogeneous pattern can herald a turn toward more severe disease.^3^ Sonographically evident third spacing of fluid has been shown to be predictive of outcome in pediatric cases of dengue hemorrhagic fever and is linked with increased mortality.^4^
